# Tissue distribution and pharmacokinetics of isoxanthohumol from hops in rodents

**DOI:** 10.1002/fsn3.3900

**Published:** 2023-12-29

**Authors:** Rie Mukai, Natsumi Hata

**Affiliations:** ^1^ Department of Food Science, Graduate School of Technology, Industrial and Social Sciences Tokushima University Tokushima Japan

**Keywords:** 8‐prenylnaringenin, bioavailability, flavanone, hop flavonoid, phytoestrogen, prenylflavonoid

## Abstract

Vegetables and fruits contain prenylflavonoids with biological functions that might improve human health. The prenylflavonoid isoxanthohumol (IXA) and its derivative, 8‐prenylnaringenin (8‐PN), have beneficial activities, including anti‐cancer effects and suppression of insulin resistance. However, their pharmacokinetic profile is unclear. Previous studies suggested flavonoids have low systemic availability and are excreted via the feces. Therefore, this study investigated the tissue distribution dynamics of high‐purity IXA (>90%) from hops administered orally, either singly (50 mg/kg body weight [BW]) or daily for 14 days (30 mg/kg BW), to mice. High‐pressure liquid chromatography demonstrated that IXA was absorbed rapidly after a single administration and reached plasma maximum concentration (*C*_max_) (3.95 ± 0.81 μmol/L) by 0.5 h. IXA was present at high levels in the liver compared with the kidney, pancreas, lung, skeletal muscle, spleen, thymus, and heart. The highest IXA level after 14 days of IXA ingestion was observed in the liver, followed by the kidney, thymus, spleen, lung, and brain. There was no significant difference in IXA accumulation in tissues between the single and multiple dose groups. Analyses of the livers of rats treated with different concentrations of IXA (112.5–1500 mg/kg BW) once a day for 28 days demonstrated that IXA accumulated dose‐dependently with a correlation coefficient of .813. The accumulation of 8‐PN was dependent on the intake period but not the intake amount of IXA (correlation coefficient −.255). In summary, IXA and 8‐PN were detected in tissues and organs up to 24 h after ingestion, suggesting that orally ingested IXA might have health benefits as a nutraceutical.

## Graphical abstract

The tissue distribution of isoxanthohumol (IXA) and its metabolite, 8‐prenylnaringenin (8‐PN), in rodents was determined using UV‐HPLC. A single administration test, a repeated administration test, and a dose‐dependent test were performed. IXA reached several tissues, accompanied by 8‐PN conversion or not, and both flavonoids may have bioaccumulation potential. The concentration of IXA in the liver was increased depending on the dosage concentration, whereas the amount of 8‐PN in the liver did not correlate with the dosage concentration.

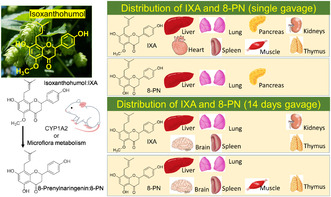

## 1 INTRODUCTION

Plant‐based foods such as vegetables, fruits, and beverages containing polyphenols might promote human health (Tufarelli et al., 2017). Flavonoids, a group of 6000 phytochemical compounds, commonly have a C6‐C3‐C6 diphenylpropane structure with some hydroxyl groups (Dias et al., 2021). Some flavonoids, termed prenylflavonoids, possess a C5 isoprenoid unit in the diphenylpropane structure (Lv et al., 2023). Fabaceae, Moraceae, and Euphorbiaceae are the three leading plant families containing prenylflavonoids (Lv et al., 2023). Recent studies have suggested that prenylflavonoids have powerful biological functions that might improve health (Chen et al., 2014; Wen et al., 2022).

**FIGURE 1:**
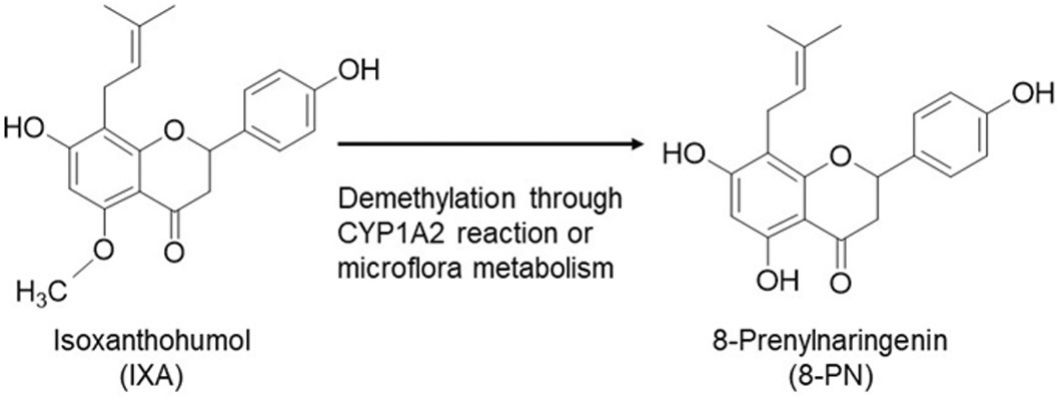
Biotransformation from IXA to 8‐PN.

Hops, *Humulus lupulus* L., are a major food ingredient containing prenylflavonoids (Stevens & Page, 2004). Xanthohumol is the most abundant prenylflavonoid (0.48% dry weight) in hops, which also contain small amounts of 4,7‐dihydroxy‐5‐methoxy‐8‐prenylflavanone (isoxanthohumol [IXA, 0.008% dry weight]) and 4,5,7‐trihydroxy‐8‐prenylflavanone (8‐prenylnaringenin [8‐PN, a 5‐*O*‐desmethyl derivative of IXA; 0.002%]) (Stevens et al., 1999). However, IXA is the most abundant prenylflavonoid in beer because most xanthohumol is converted to IXA by heating during beer production (Jan F. Stevens et al., 1999). In addition, xanthohumol is also converted to IXA under acidic conditions (Nikolic et al., 2005), such as in the stomach environment. IXA is further converted to 8‐PN by cytochrome P450 1A2 in the liver (Guo et al., 2006) or by intestinal microflora such as *Eurobacterium limosum* (Possemiers et al., 2005) (Figure 1). These previous reports suggest that IXA is the major contributor to the bioactivity of hops and beer and is a precursor of 8‐PN. 8‐PN is a potent phytoestrogen (Milligan et al., 1999) that maintains bone mass (Komrakova et al., 2019; Luo et al., 2014) and skeletal muscle mass (Mukai et al., 2016) in ovariectomized animals. The biological activity of IXA to promote health has been reported (Żołnierczyk et al., 2015). IXA functions as an anti‐cancer phytochemical by inhibiting the growth and/or proliferation of cultured cells, including ovarian cells (A2780), colon cancer cells (HT‐29 and SW620), and breast cancer cells (MCF‐7) (Bartmańska et al., 2018; Miranda et al., 1999; Seliger et al., 2019). Animal studies demonstrated that IXA prevented fat‐weight gain, affected fecal metabolite components, and suppressed insulin resistance in mice fed a high‐fat diet (Fukizawa, Yamashita, Fujisaka, et al., 2020; Fukizawa, Yamashita, Wakabayashi, et al., 2020; Yamashita et al., 2020). IXA also inhibited the differentiation of 3T3L1 adipocytes and suppressed sterol regulatory element‐binding protein activity in Huh‐7 cells (Inoue et al., 2018; Kiyofuji et al., 2014).

The rational selection of flavonoid analogs for advancement into clinical development requires a robust knowledge of their preclinical efficacy and biochemical mechanisms, as well as their absorption, delivery, distribution, metabolism, and excretion rates. Pharmacokinetic studies of hop extract, including xanthohumol, 8‐PN, and 6‐prenylnaringenin, were performed in menopausal women (van Breemen et al., 2014). Three different dosing concentrations were administered, and the plasma concentrations of IXA and 8‐PN were high depending on the concentration of the dose (van Breemen et al., 2014). The serum concentration–time curve of these phytochemicals has two or three peaks, suggesting they were incorporated into the blood and cycled through the enterohepatic circulation (van Breemen et al., 2014), similar to other flavonoids (Chen et al., 2022; Naeem et al., 2022). A study of the enantiospecific pharmacokinetics of pure IXA estimated that the bioavailability was 4%–5% (Martinez et al., 2014) and the rate of excretion into the feces was approximately 47% (Possemiers et al., 2008). These data follow the general characterization of flavonoids as having low systemic availability (Chen et al., 2022; Naeem et al., 2022). IXA in the blood reached the liver and underwent phase II metabolism, including glucuronidation and sulfation similar to other flavonoids (Buckett et al., 2022; Martinez & Davies, 2015). Other specific metabolisms of IXA in the liver include the demethylation of IXA to 8‐PN by CYP1A2 and the hydroxylation of the prenyl group to cis and trans alcohols by CYP2C19 and CYP2C8 (Guo et al., 2006; Nikolic et al., 2005). Although a previous study reported the tissue distribution of IXA by detecting IXA and 8‐PN in human breast tissues in women who were administered a hop supplement (Bolca et al., 2010), the detailed tissue distribution of IXA has not been reported. The tissue distribution dynamics of IXA might have important implications for the clinical and nutritional applications of IXA. To evaluate the tissue distribution dynamics of IXA, we conducted animal experiments with high‐purity IXA. In this study, we traced the delivery of IXA to tissues over time after a single dose and determined the change in IXA and 8‐PN tissue accumulation after a prolonged intake period or increased dosage concentration of IXA.

## 2 METHODS AND MATERIALS

### 2.1 Materials

An IXA‐enriched product named Isoxanthoflav (Hopsteiner, Mainburg, Germany) with a purity of IXA over 90% was used for the study and was the same product used in a previous report (Fukizawa, Yamashita, Fujisaka, et al., 2020). Quercetin‐3,3′,4′,7‐tetramethyl ether (QTM) was purchased from Extrasynthase (1074, Lyon, France, >90% purity, CAS No. 1245‐15‐4). 8‐PN (>95% purity) was synthesized by our group (Kawamura et al., 2012).

### 2.2 Animal experiments

Experimental protocols were approved by the Committee on Animal Experiments at Tokushima University (#T2020‐121 for mice) or the Ethics Committee of Animal Experiments at the BioSafety Research Center Inc. (19‐0185A for rats). All efforts were made to minimize harm and the number of animals used. All surgeries were performed after euthanasia with anesthesia. A mixed anesthetic agent containing 0.3 mg/kg body weight (BW) of medetomidine (801040, Nippon Zenyaku Kogyo Co., Ltd., Fukushima, Japan), 4.0 mg/kg BW of midazolam (871124, Astellas Pharma Inc., Tokyo, Japan), and 5.0 mg/kg BW of butorphanol (Meiji Seika Pharma Co., Tokyo, Japan VETIF546) was dissolved in saline (873311, Otsuka Pharmaceutical Co., Ltd., Tokyo, Japan) and administered intraperitoneally to mice; 3% isoflurane as a gaseous anesthesia was used for rats.

### 2.3 Dosage information

All animals were dosed with IXA by gavage. Experimental conditions, including ingestion timing, frequency, and duration, are described in each experimental section. The dosage concentration in mice was determined with reference to previous similar research (Mukai et al., 2013; Tanaka et al., 2022). We converted animal doses to human equivalent doses based on the body surface area according to the guidelines, ‘Guidance for Industry Estimating the Maximum Safe Starting Dose in Initial Clinical Trials for Therapeutics in Adult Healthy Volunteers’ (Alert, 2005). The dosage concentration used in this study was 30 mg/kg BW and 50 mg/kg BW in mice, and the human equivalent doses were calculated as 2.4 and 4.1 mg/kg BW, respectively. The dose conditions were achievable via available supplements. For rat experiments, we administered 112.5–1500 mg/kg BW to rats, and the human equivalent dose was 1.81–241.9 mg/kg BW. Because rat experiments were performed to determine the accumulation limit of IXA in the liver, the dose was set higher than that obtained from the diet or supplements.

### 2.4 Single‐gavage administration of IXA to mice

Six‐week‐old male C57/BL6 mice (initial BW: 19–24 g; Japan SLC, Shizuoka, Japan) were housed in a room maintained at 23–26°C with a 12‐h light–dark cycle. During the 1‐week acclimatization period, they were allowed free access to a commercial diet (Oriental Yeast Co., Tokyo, Japan) and water. Two days before the experiment, they were fed AIN‐93M (Oriental Yeast Co.). Before drug administration, they were fasted for 20 h, but had free access to water. IXA dissolved in 3% hydroxypropyl‐β‐cyclodextrin and 5% ethanol (Fujifilm; Wako Pure Chemical Corp., Osaka, Japan) was named the injection solution and administered (50 mg/kg BW) to mice intragastrically using a gastric feeding tube (AS ONE, Osaka, Japan). Blood, brain, thymus, heart, lung, liver, spleen, pancreas, kidney, and gastrocnemius muscle were collected 0.5, 1, 2, 4, 8, 12, 24, and 48 h after administration. We also set up a control group, and mice in this group were sacrificed just before we started administering IXA at 0 h. At 4 h after administration, mice were allowed free access to AIN‐93 M. Plasma was isolated by centrifugation at 17 × 10^3^ m/s^2^ (5911, Kubota, Tokyo, Japan) for 5 min at 4°C. All tissues were dissected, weighed, and immediately frozen in liquid nitrogen. All samples were stored at −80°C until analysis.

### 2.5 Daily gavage administration of IXA to mice for 14 days

Animal species, age, sex, initial BW, feeding conditions, and dosage method were the same as those for the single gavage administration test, but the dose was 30 mg/kg BW. We did not re‐establish a control group for this test because it was performed under the same experimental conditions as the single gavage test mentioned above. Daily gavage administration without fasting was performed at 12:00 pm for 14 days. The final BW was 21–26 g. Blood, brain, thymus, heart, lung, liver, spleen, kidney, and gastrocnemius muscle were collected 24 h after the final administration. The sample collection method was the same as that for the single gavage administration test.

### 2.6 Intragastric IXA administration at various concentrations to rats for 28 days

Six‐week‐old female Sprague–Dawley rats (Charles River Laboratories Japan, Inc., Yokohama, Japan) were housed in a room maintained at 20–26°C on a 12‐h light–dark cycle. The initial BW of rats was 176–179 g. They were allowed free access to a commercial diet (CRF‐1) and water during the experiment. IXA dissolved in injection solution was administered intragastrically to rats (112.5, 225, 360, 450, and 1500 mg/kg BW) daily for 28 days using a gastric feeding tube. We also set up a control group to which the injection solution was administered. The final BW of rats was 231–241 g. The liver was collected the day after the last administration. The rats were fasted for 16–24 h before they were euthanized, but they had free access to water. Liver samples were stored at −80°C until analysis.

### 2.7 Sample preparation for high‐performance liquid chromatography (HPLC) analysis

Plasma samples were deconjugated by β‐glucuronidase type H‐1 (which has β‐glucuronidase and sulphatase activity; Sigma, St. Louis, MO, USA; G0751) to convert aglycon from conjugated metabolites of IXA and 8‐PN, as described in a previous report (Y. Tanaka et al., 2022). Briefly, 100 μL of plasma was mixed with 900 μL of 0.1 mol/L sodium acetate buffer (pH 4.7), 200 μL of 50 mmol/L ascorbic acid, and 1 mL of 1.67 μkat/100 μL of β‐glucuronidase type H‐1, and then incubated for 30 min at 37°C. Then, 2.4 nmol of QTM was added to the samples as the internal standard. The liberated aglycone was extracted using ethyl acetate (Kanto Kagaku, 14,029‐2B).

Tissue sample deconjugation was performed as described in a previous report with slight modifications (Tanaka et al., 2022). Tissues were homogenized in phosphate‐buffered saline (pH 7.4), which was nine times the tissue volume. The tissue homogenate was incubated in 50 mmol/L ascorbic acid (one‐fifth the volume of phosphate‐buffered saline) and 1.67 μkat/100 μL of β‐glucuronidase type H‐1 in 0.1 mol/L sodium acetate buffer (pH 4.7, nine times the volume of the sample) for 2 h at 37°C. Then, 2.4 nmol of QTM was added to samples as an internal standard. The supernatant was isolated by centrifugation at 17 × 10^3^ m/s^2^ (Kubota) for 5 min at 4°C. Then, methanol (Kanto Kagaku, 25183‐2B) was added to the pellets, and they were shaken for 1 min. The methanol layer and pellets were separated by centrifugation at 17 × 10^3^ m/s^2^ (Kubota, RY1071‐A000) for 5 min at 4°C. Methanol extraction was repeated three times. Finally, methanol extracts were mixed with the supernatant (the composition of the mixture was 30% methanol) and then applied to an Inert Sep C8 (200 mg/3 mL; GL Sciences, Tokyo, Japan; 5010‐61082) cartridge. The cartridge was preconditioned with 6 mL of 2% acetic acid in methanol, and then 9 mL of 2% acetic acid in water. The extracted tissue was passed through the cartridge, and it was washed with 5 mL of 2% acetic acid in 30% methanol *aq*. The flavonoids were eluted with 10 mL of 2% acetic acid in methanol.

The plasma and tissue extracts were evaporated using a Centrifugal Evaporator (EYERA, Tokyo, Japan, CVE‐3000). The samples were stored at −80°C until the next day. Then, the samples were dissolved in 120 μL of a mobile phase solution and divided into two test tubes. The first sample was injected into the HPLC with an ultraviolet detection system (Shimadzu, Kyoto, Japan). The second tube containing 60 μL of the test sample was mixed with 1.2 nmol of IXA and 8‐PN to identify each compound by the increased peaks at retention time on standard compounds caused by adding these compounds.

### 2.8 HPLC analyses to determine IXA and 8‐PN concentrations

IXA and 8‐PN concentrations were determined by their respective standard curves using an internal standard method. A Cadenza 3‐μm CD‐C18 HPLC column (CD005, 150 × 4.6 mm; Imtakt Corporation, Kyoto, Japan) was equipped for HPLC (*λ* = 295 nm). The mobile phase consisted of methanol:water:acetic acid (60:35:5, v/v/v). The flow rate was set at 1.0 mL/min. We confirmed that there were no apparent peaks comparable with IXA or 8‐PN in chromatograms of tissue from non‐fed mice (Supplemental Data: Figure S1), suggesting there was no interference from peaks that overlapped with the peaks used in the determination. Because clear peaks over 4.5 pmol/sample were detected for IXA and 8‐PN by visual evaluation, we determined that these concentrations were the peak limit of detections for each flavonoid. We applied calibration curves in the IXA/QTM concentration ratio range of 0.007–35.1 (*R* ^2^ = .9955). The calibration curve in the 8‐PN/QTM concentration ratio range of 0.039–15.5 showed linearity (*R* ^2^ = .9994). The tested recovery rate and inter‐ and intra‐day coefficients of variation are listed in Table 1.

**TABLE 1:** HPLC validation.

	IXA	8‐PN
Plasma recovery^†^ (%)	95.1 ± 5.55	94.6 ± 5.42
Liver recovery^†^ (%)	87.3 ± 6.30	80.5 ± 2.32
Intra‐day CV^‡^ (%)	0.35	1.50
Inter‐day CV^§^ (%)	0.95	0.94

Abbreviations: 8‐PN, 8‐prenylnaringenin; CV, coefficient of variation; IXA, isoxanthohumol.

^†^ Values are presented as the mean ± standard deviation (*n* = 3).

^‡,§^CVs were determined by comparing different injections on the same day (*n* = 3, intra‐day) or different injections on different days (*n* = 3, inter‐day).

### 2.9 Statistical analyses

Data from animal experiments are presented as the mean ± standard error of the mean (SEM). Statistical analyses were performed using Excel Toukei version 7.0 (Esumi, Tokyo, Japan). The data in Table 3 and Figure 3 were analyzed by one‐way analysis of variance followed by the Tukey–Kramer multiple comparison test. For Table 4, we used the Mann–Whitney *U*‐test to compare the amount of tissue accumulation between a single‐dose study and a 14‐day dose study. The correlation coefficient between dose‐concentration and accumulation in Figure 3 was analyzed by Pearson's correlation coefficient. *p‐*Values < .05 were considered statistically significant.

## 3 RESULTS

### 3.1 Pharmacokinetics and time course of tissue distribution of IXA and its metabolite, 8‐PN, in mice after a single ingestion of IXA

**FIGURE 2:**
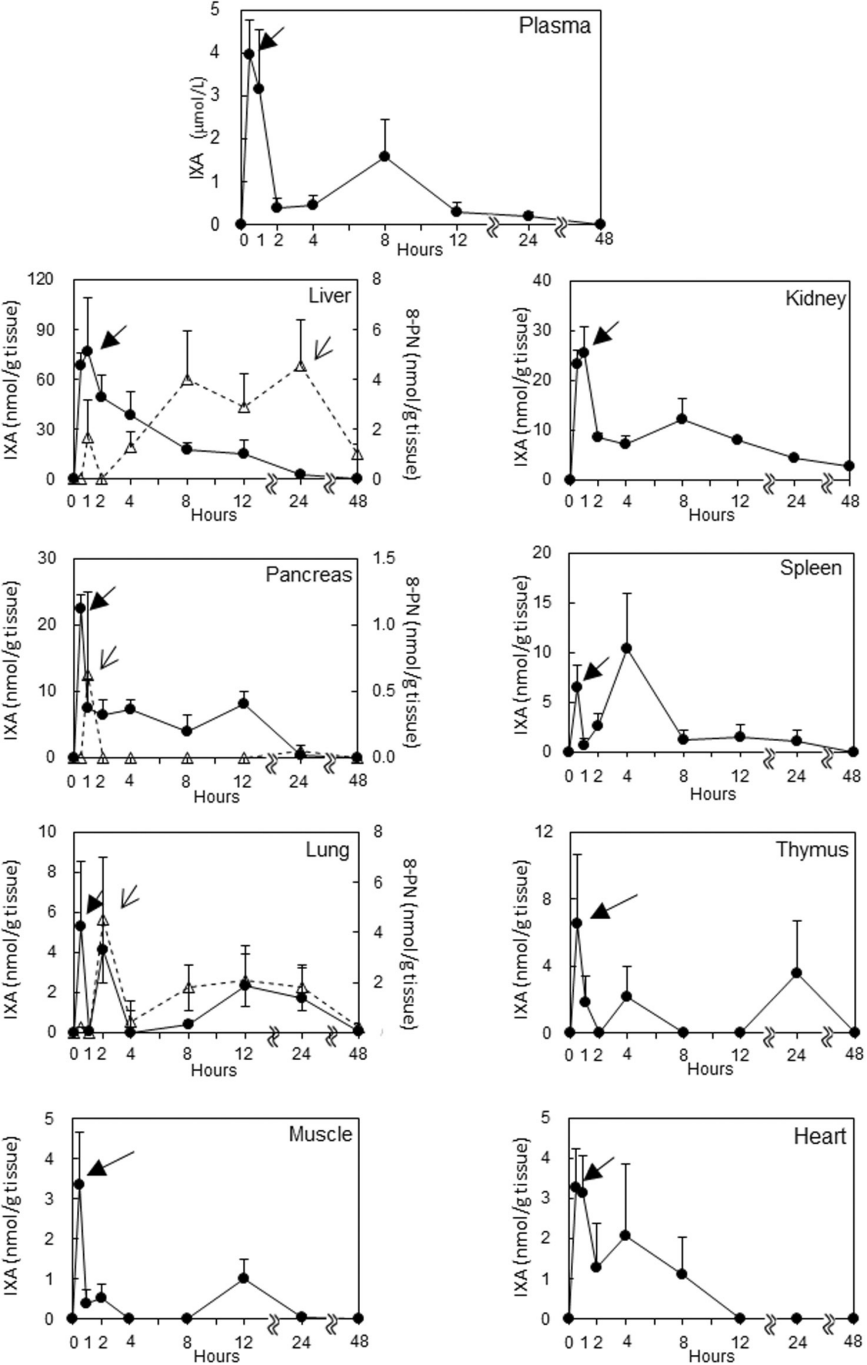
IXA and 8‐PN concentrations in plasma and tissues after a single intragastric ingestion of IXA to mice (50 mg/kg BW). IXA (vertical axis on the left line) and 8‐PN (vertical axis on the right line) values determined by HPLC are the sum of aglycone and conjugated metabolites extracted from tissues with deconjugation treatment. Closed circle: IXA; open triangle: 8‐PN. The closed triangular arrows indicate the *C*_max_ of IXA, and the open triangular arrows indicate the *C*_max_ of 8‐PN. The data are presented as the mean + SEM, *n* = 4. Spleen values were measured at 4 h (*n* = 3).

We determined the concentration of IXA and its metabolite, 8‐PN, after a single ingestion of IXA at 50 mg/kg BW (Figure 2). In the plasma, IXA reached its maximum concentration (*C*_max_) of 3.95 ± 0.81 μmol/L 0.5 h after administration. IXA was increased further 8 h after administration. At 48 h after administration, IXA was not detected. 8‐PN was not observed in the plasma during the experiment (data not shown). The pharmacokinetic parameters of IXA are shown in Table 2. The time to maximum concentration (T_max_) was 0.6 h after ingestion, and the half‐life of IXA in the mean elimination phase (T_1/2_) was 1.43 h. The liver had a significantly higher *C*_max_ for IXA among the tested tissues (Table 3). The highest *C*_max_ was observed in the liver, followed by the kidney, pancreas, spleen, thymus, lung, muscle, and heart (Figure 2 and Table 3). IXA was not detected in the brain (data not shown). The highest peak of IXA was reached 0.5–1 h after ingestion in all tissues except the spleen. The second peak was detected at 2 h in the lung; 4 h in the spleen, thymus, and heart; 8 h in the kidney; and 12 h in the pancreas and skeletal muscle. IXA was detected in the liver (0.14 ± 0.12 nmol/g tissue), kidney (2.67 ± 0.41 nmol/g tissue), and lung (0.05 ± 0.04 nmol/g tissue) up to 48 h after ingestion. 8‐PN was also detected in the liver, pancreas, and lung. The ratios of the area under the mean concentration–time curve (AUC) for 8‐PN to that of IXA in the liver, lung, and pancreas were 26%, 109%, and 1%, respectively.

**TABLE 2:** IXA pharmacokinetic parameters after a single oral administration of IXA (50 mg/kg BW) to mice.

Parameter	IXA
*C*_max_ (μM)	4.0 ± 0.8
AUC_24h_ (μmol h/L)	18.2
*T*_max_ (h)	0.6 ± 0.1
*T*_1/2_ (h)	1.4

*Note*: IXA (50 mg/kg BW) was administered to mice once intragastrically (*n* = 4). The plasma concentration was analyzed by HPLC. *C*_max_ and *T*_max_ data are presented as the mean ± SEM. The AUC and half‐life of IXA in *T*_1/2_ were calculated using the mean (*n* = 5) at each time point.

Abbreviations: AUC, area under the curve; BW, body weight; IXA, isoxanthohumol.

**TABLE 3:** IXA and 8‐PN accumulation parameters in a single‐ingestion test.

	*C*_max_ (nmol/g of tissue)		AUC (0–48 h)		
	IXA	8‐PN	IXA	8‐PN	8‐PN/IXA (%)
Liver	76.2 ± 32.4^a^	4.6 ± 1.8	537.2	139.4	26.0
Kidney	25.5 ± 5.3^a,b^	n.d.	292.6	–	–
Pancreas	22.4 ± 2.1^b,c^	0.6 ± 0.5	137.1	1.3	1.0
Spleen	6.5 ± 2.2^b,c^	n.d.	75.1	–	–
Thymus	6.5 ± 4.1^b,c^	n.d.	75.5	–	–
Lung	5.3 ± 3.3^b,c^	4.5 ± 2.5	61.2	66.9	109.3
Muscle	3.4 ± 1.3^b,c^	n.d.	11.7	–	–
Heart	3.3 ± 1.0^b,c^	n.d.	16.5	–	–
Brain	n.d.	n.d.	0	–	–

*Note*: *C*_max_ of IXA and 8‐PN in each tissue after the single ingestion of IXA. IXA in the liver and kidneys was measured at 1 h, and at 0.5 h in other tissues. 8‐PN was measured at 24 h in the liver, at 1 h in the pancreas, and at 2 h in the lung. Values are extracted from Figure 2. Data are presented as the mean ± SEM (*n* = 4). Different symbols indicate statistically significant differences between the *C*_max_ of IXA in each tissue (*p* < .05).

Abbreviations: 8‐PN, 8‐prenylnaringenin; IXA, isoxanthohumol; n.d., not detectable.

### 3.2 Tissue distribution of IXA and its metabolite, 8‐PN, after repeated administration to mice for 14 days

The highest IXA level after 14 days of repeated ingestion in mice was observed in the liver, followed by the kidney, thymus, spleen, lung, and brain (Table 4). The highest 8‐PN level was observed in the lungs, followed by the liver, brain, spleen, thymus, and muscle. IXA and 8‐PN were not detected in the plasma and heart (Table 4). However, statistical analysis using the Mann–Whitney *U*‐test to compare the level of accumulation between the 14‐day repeat dose test and the single dose test did not indicate a significant difference. However, flavonoid accumulation after repeated administration tended to be higher compared with that at 24 h after a single ingestion, except for 8‐PN in the liver and IXA in the plasma.

**TABLE 4:** IXA and 8‐PN accumulation in mice administered IXA after a 14‐day repeated‐ingestion test and at 24 h after a single‐ingestion test.

	nmol/g of tissue, μmol/L of plasma			
	14‐day repeated‐ingestion test (30 mg/kg BW)		Single‐ingestion test (at 24 h)^†^ (50 mg/kg BW)	
Tissue	IXA	8‐PN	IXA	8‐PN
Liver	8.6 ± 2.9	2.1 ± 0.4	3.2 ± 1.3	4.6 ± 1.8
Kidney	5.8 ± 1.0	n.d.	4.6 ± 0.3	n.d.
Thymus	4.3 ± 3.8	0.6 ± 0.5	3.6 ± 3.1	n.d.
Spleen	3.4 ± 2.0	0.6 ± 0.6	1.1 ± 1.0	n.d.
Lung	3.2 ± 2.8	3.9 ± 3.5	1.7 ± 1.5	1.8 ± 0.9
Brain	0.2 ± 0.1	0.8 ± 0.7	n.d.	n.d.
Muscle	n.d.	0.1 ± 0.0	0.1 ± 0.1	n.d.
Heart	n.d.	n.d.	n.d.	n.d.
Plasma	n.d.	n.d.	0.2 ± 0.1	n.d.

*Note*: IXA and 8‐PN values are the sum of aglycone and conjugated metabolites obtained by HPLC analysis with deconjugation treatment. The data are presented as the mean ± SEM (*n* = 4). We analyzed significant differences for each compound between the 14‐day repeated‐ingestion test and single‐ingestion test (*p* > .05, Mann–Whitney *U*‐test), but there are no differences between them.

Abbreviations: 8‐PN, 8‐prenylnaringenin; BW, body weight; IXA, isoxanthohumol; n.d., not detectable.

^†^ Data of the single‐ingestion test are the same as the data presented in Figure 2.

### 3.3 Liver accumulation of IXA after repeated administration of various doses for 28 days in rats

**FIGURE 3:**
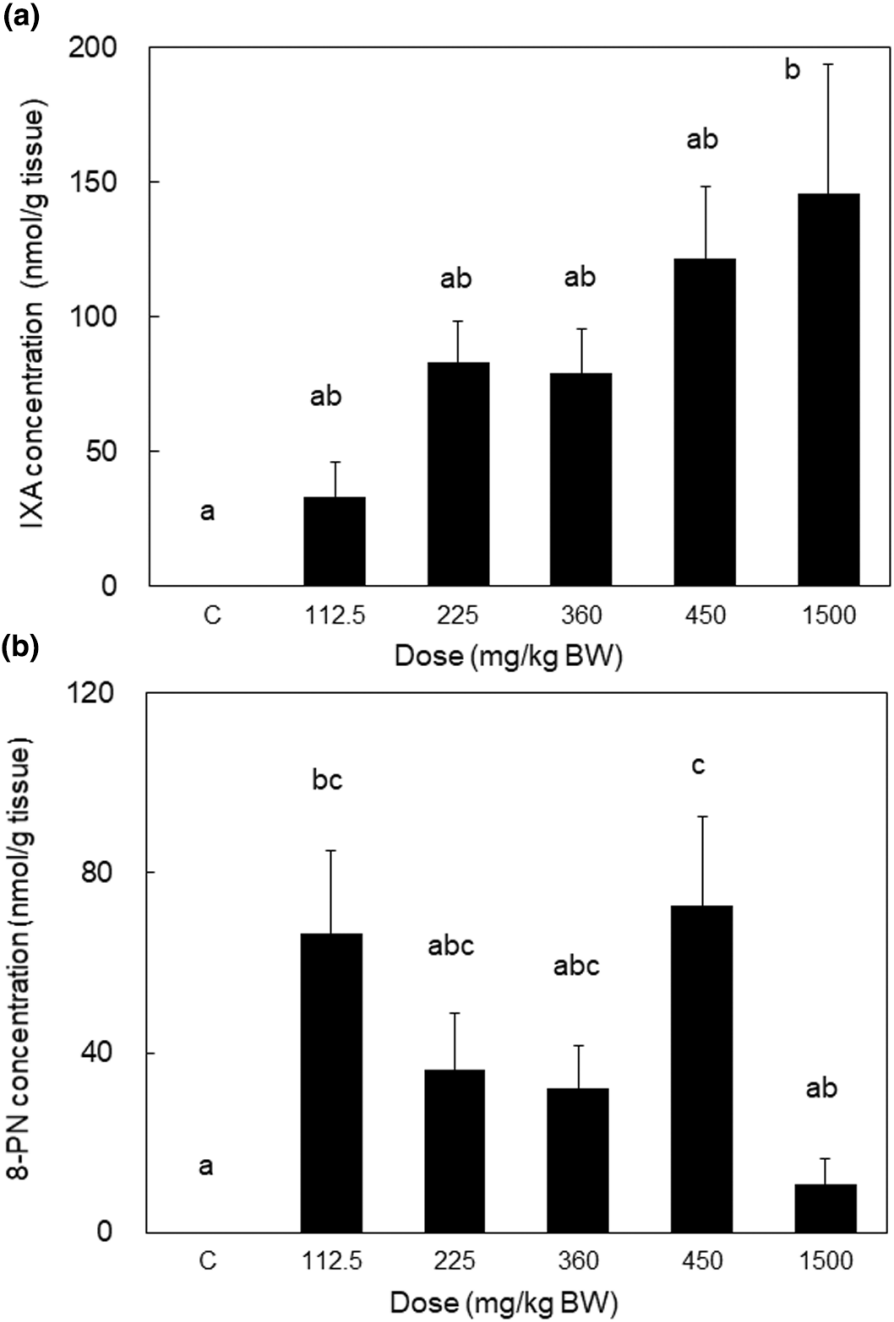
IXA and 8‐PN accumulation in the liver after intragastric IXA administration of the indicated dose to rats for 28‐days. IXA (a) and 8‐PN (b) values determined by HPLC are the sum of aglycone and conjugated metabolites extracted from tissues with deconjugation treatment. The data are presented as the mean ± SEM (*n* = 7). Different letters indicate statistically significant differences among each dose (*p* < .05).

IXA accumulation was increased in rats as the administered dose increased (112.5–1500 mg/kg BW) (Figure 3). The correlation coefficient between dose‐concentration and accumulation of IXA was .813, whereas it was −.255 for 8‐PN. The amount of 8‐PN was approximately two times higher than that of IXA in rats that ingested 112.5 mg/kg IXA, and it was approximately 7.4% of the 8‐PN levels compared with those of IXA in rats that received IXA 1500 mg/kg.

## 4 DISCUSSION

This study investigated the tissue distribution of high‐purity IXA using animal experiments to evaluate the use of IXA as a nutraceutical. IXA was absorbed rapidly and then reached the *C*_max_ in plasma within 1 h. IXA was distributed to several tissues and organs, including the liver, kidney, pancreas, lung, skeletal muscle, spleen, thymus, and heart. The pharmacokinetic parameters in each tissue were independent of the pharmacokinetic parameters in the plasma. The elimination phase was clearly different between tissues. A longer intake period and increased intake amount resulted in a higher accumulation of IXA (Table 4 and Figure 3). The accumulation of 8‐PN was dependent on the intake period but not the intake amount of IXA. The distribution of IXA in tissues was similar to that of other flavonoids (Mukai et al., 2013; Tanaka et al., 2022).

The tissue distribution of gliceolin‐1, a prenylated isoflavone, and 5,7‐dimetoxy flavone, a methylated flavone, after a single ingestion was reported previously (Bei & An, 2016; Zhang et al., 2021). The *C*_max_ of these flavonoids in the liver and kidney were higher than in other tissues, similar to our results. Previous reports demonstrated that IXA underwent phase II metabolism (Buckett et al., 2022) and that these metabolites were excreted into the urine (Martinez & Davies, 2015). These studies suggested that IXA is a substrate for drug metabolizing enzyme systems (Chen et al., 2022; Naeem et al., 2022), especially as IXA is primarily transported and distributed into the liver and kidney, which contribute to drug metabolism (Murota et al., 2018). The *C*_max_ in the pancreas was relatively higher than that in other tissues except for the liver, and accumulation was maintained until 12 h post‐ingestion. Although previous studies have reported the presence of flavonoids in the pancreas (Takumi et al., 2011; Zhang et al., 2018), the amount was lower compared with other tissues. However, IXA accumulates in the pancreas in high amounts, which is important because it can influence digestive enzymes and insulin secretion that might result in anti‐diabetic effects (Yamashita et al., 2020) related to obesity (Fukizawa, Yamashita, Wakabayashi, et al., 2020), which is of interest in the field of nutritional pathology. However, IXA did not appear in the brain after a single dose; therefore, a continuous intake of IXA will be needed to achieve its pharmacological effect as a sleeping aid (Benkherouf et al., 2019).

CYP1A2 is an enzyme that converts IXA to 8‐PN in the liver (Karabin et al., 2015). Although CYP1A2 is mainly expressed in the liver, CYP1A2 expression in the lungs was reported by Gerges and El‐Kadi (2023). Therefore, 8‐PN accumulation in the liver and lungs depends on the metabolic conversion of IXA to 8‐PN in these tissues. Other than the liver and lungs, there is no information about CYP1A2 expression, and thus, converted 8‐PN may be transported from these tissues to other tissues. However, Figure 3 shows that there was no correlation between the dose of IXA and 8‐PN accumulation. It is difficult to explain this phenomenon by considering only CYP1A2 expression in the liver. It was reported that 40% of human subjects produced high levels of 8‐PN from IXA, which was dependent on their intestinal microflora (Karabin et al., 2015). Although we did not analyze the intestinal microflora of animals in our study, microflora variation was observed in the mice (Frolinger et al., 2019). We also tried to confirm the presence of 6‐PN in the samples analyzed in this study by using a reference standard, but it was not detected (data not shown). Other studies revealed that 6‐PN was not detected in the plasma of rats (Legette et al., 2012) or human subjects (Legette et al., 2014) who ingested xanthohumol, but was confirmed in feces (Bai et al., 2022). Although we did not analyze the feces, our data inferred that there was minimal to no 6‐PN circulation in the body after IXA ingestion.

IXA in plasma was detected until 24 h after ingestion (Figure 2), in agreement with a previous report (Martinez & Davies, 2015). Because the IXA concentration in several tissues, after ingestion for 14 days, was higher than that 24 h after a single ingestion, the ingestion of 30 mg/kg BW led to a daily excretion capacity larger than normal, resulting in the accumulation of IXA. IXA and 8‐PN accumulation increased as the dosing period increased (Table 4). Our data suggest the bioaccumulation potential of IXA, and it will be important to establish an effective dose range of IXA suitable for health promotion. Our previous studies suggested that prenylflavonoids showed unique absorption and elimination patterns compared with aglycone flavonoids (Mukai et al., 2012, 2013). Generally, flavonoids efflux from cells by ATP‐binding cassette transporters (Mukai et al., 2012). The prenylation of flavonoids avoids the efflux of these transporters, resulting in the increased accumulation of prenylflavonoids in cells (Mukai et al., 2012, 2013). Although prenylation of the flavonoid mother structure increased the uptake of these compounds into enterocytes, this structural modification inhibited transportation to the basolateral side (Mukai et al., 2013). The lipophilic structure related to prenylation may contribute to the retention of flavonoids in cells because of a hydrophobic interaction with proteins (Pang et al., 2007). It was also reported that prenylation of flavonoids enhanced its interaction to human serum albumin, which may have the advantage of extending the circulation period; thus, serum albumin might be a candidate carrier for IXA circulating in the blood and through organs (Tanaka et al., 2022). In a clinical study, IXA glucuronides were detected in the urine from 24 h post‐ingestion to 120 h post‐ingestion (Martinez & Davies, 2015). A pharmacokinetic study of icaritin, a flavonol‐type prenylflavonoid, showed its rapid conjugation and slow elimination from the plasma (Huang et al., 2019). Taken together, these data suggest IXA circulates between the blood and certain organs for several days and then is eliminated slowly, resulting in bioaccumulation potential.

Bioavailability properties are species‐specific (Bai et al., 2020; Bian et al., 2022); data from mice cannot be directly extrapolated to humans, and obtaining biopsy samples from humans is extremely difficult; therefore, our data are a valuable reference for investigating the health benefits of IXA in clinical trials. An experimental limitation was that the intestinal flora was not fully controlled, although 8‐PN is an intestinal bacterial metabolite (Possemiers et al., 2005, 2008). In this study, the conjugated metabolites were hydrolyzed by the deconjugation reaction, and then the total concentration (subtotal of aglycone and conjugated metabolites) was analyzed. Therefore, it was not possible to confirm the impact of metabolite composition on the IXA concentration in each tissue. It should also be noted that extending the intake period complicates metabolite composition (Tanaka et al., 2019) and may alter the phenotype associated with IXA biological activity. After oral ingestion, xanthohumol was found in epithelial cells in the gastrointestinal tract (Wolff et al., 2011) and in breast tissues (Karabin et al., 2015). Because the metabolites of xanthohumol are mainly excreted into the feces (Bai et al., 2022), future studies should detect IXA in the digestive tract and feces for comparison purposes. Further study will be needed to clarify these issues and improve our knowledge of IXA tissue distribution so it can be used as a nutraceutical.

In the present study, we investigated the characteristics of the tissue distribution of IXA. The changes in IXA concentration in each tissue and plasma were independent of each other, and thus, our data provide information about optimal timepoints at which biological effects are exerted in a target tissue. Because extending the intake period and increasing the intake concentration led to an increase in tissue accumulation (Table 4 and Figure 3), we propose that the conditions for IXA intake as a dietary supplement should consider the side effects caused by excessive accumulation. IXA is converted from xanthohumol by a heating process and becomes the major prenylflavonoid in hops (Mudura & Coldea, 2015). Therefore, beer, including non‐alcoholic types, nutritional supplement tablets, or the crude extract from hops subjected to heating, can be used as a source of IXA supplementation. Our data showed that IXA and 8‐PN were detectable up to 24 h after ingestion, suggesting that once‐daily orally ingested IXA can exert health benefits as a nutraceutical.

## AUTHOR CONTRIBUTIONS

**Rie Mukai:** Conceptualization (lead); data curation (lead); formal analysis (lead); investigation (lead); methodology (lead); project administration (lead); supervision (lead); validation (lead); visualization (lead); writing – original draft (lead); writing – review and editing (lead). **Natsumi Hata:** Investigation (supporting); visualization (supporting); writing – original draft (supporting).

## CONFLICT OF INTEREST STATEMENT

The authors declare no conflict of interest.

## ETHICS STATEMENT

Experimental protocols were approved by the Committee on Animal Experiments at Tokushima University (#T2020‐121 for mice) or the Ethics Committee of Animal Experiments at the BioSafety Research Center Inc. (19‐0185A for rats).

## INFORMED CONSENT

This study did not include the clinical study that needs informed consent.

## Supporting information


Figure S1.

## Data Availability

The data underlying this article will be shared upon reasonable request to the corresponding author.
