# Supplementary figures and images for: Tissue distribution and pharmacokinetics of isoxanthohumol from hops in rodents
Source: Food Sci Nutr. 2023 Dec 29;12(3):2210–9. doi: 10.1002/fsn3.3900 (PMC10916623; doi:10.1002/fsn3.3900)

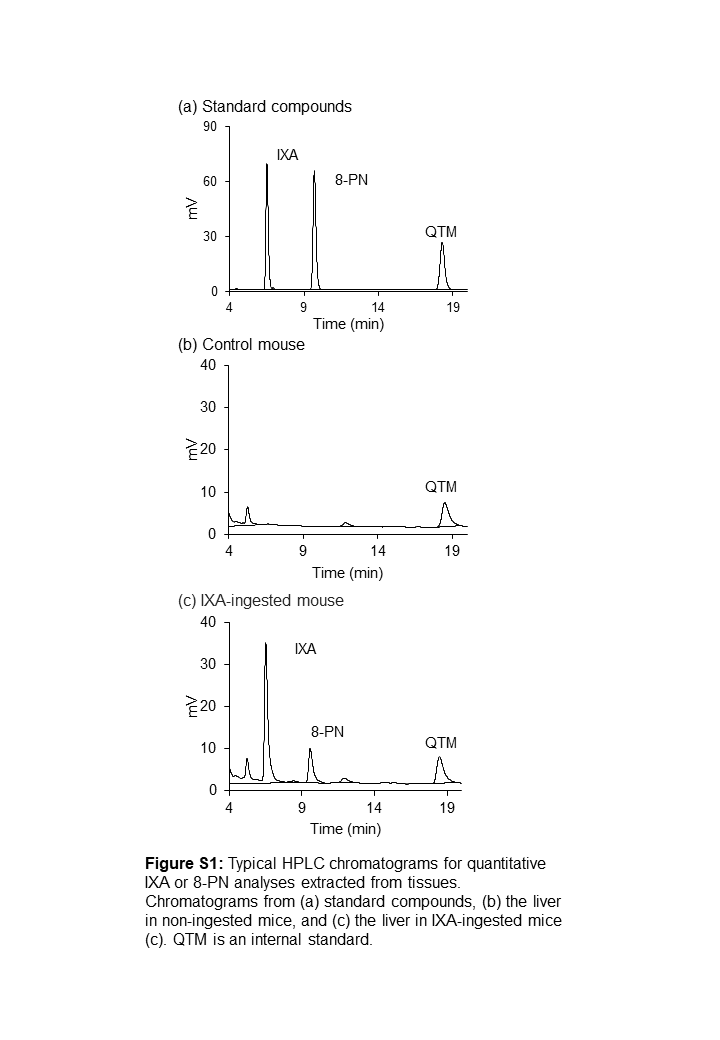

Supplement: Supplementary file 1 — Figure S1. [file FSN3-12-2210-s001.tif]
